# Investigating the Hepatitis E Virus (HEV) Diversity in Rat Reservoirs from Northern Italy

**DOI:** 10.3390/pathogens13080633

**Published:** 2024-07-29

**Authors:** Luca De Sabato, Marina Monini, Roberta Galuppi, Filippo Maria Dini, Giovanni Ianiro, Gabriele Vaccari, Fabio Ostanello, Ilaria Di Bartolo

**Affiliations:** 1 Department of Food Safety, Nutrition and Veterinary Public Health, Istituto Superiore di Sanità, Viale Regina Elena, 299, 00161 Rome, Italy; luca.desabato@iss.it (L.D.S.); giovanni.ianiro@iss.it (G.I.); gabriele.vaccari@iss.it (G.V.); ilaria.dibartolo@iss.it (I.D.B.); 2 Department of Veterinary Medical Sciences, University of Bologna, Via Tolara di Sopra, 50, Ozzano dell’Emilia, 40064 Bologna, Italy; roberta.galuppi@unibo.it (R.G.); filippomaria.dini@unibo.it (F.M.D.); fabio.ostanello@unibo.it (F.O.)

**Keywords:** hepatitis E virus, reservoir, rat, zoonoses, HEV-C1, Italy

## Abstract

Hepatitis E virus belonging to the *Rocahepevirus ratti* species, genotype HEV-C1, has been extensively reported in rats in Europe, Asia and North America. Recently, human cases of hepatitis associated with HEV-C1 infection have been reported, but the zoonotic nature of rat-HEV remains controversial. The transmission route of rat-HEV is unidentified and requires further investigation. The HEV strains of the *Paslahepevirus balayani* species, belonging to the same *Hepeviridae* family, and including the zoonotic genotype HEV-3 usually found in pigs, have also sporadically been identified in rats. We sampled 115 rats (liver, lung, feces) between 2020 and 2023 in Northeast Italy and the HEV detection was carried out by using Reverse Transcription PCR. HEV RNA was detected in 3/115 (2.6%) rats who tested positive for HEV-C1 strains in paired lung, intestinal contents and liver samples. Overall, none tested positive for the *Paslahepevirus balayani* strains. In conclusion, our results confirm the presence of HEV-rat in Italy with a prevalence similar to previous studies but show that there is a wide heterogeneity of strains in circulation. The detection of HEV-C1 genotype of *Rocahepevirus ratti* species in some human cases of acute hepatitis suggests that HEV-C1 may be an underestimated source of human infections. This finding, with the geographically widespread detection of HEV-C1 in rats, raises questions about the role of rats as hosts for both HEV-C1 and HEV-3 and the possibility of zoonotic transmission.

## 1. Introduction

The hepatitis E virus (HEV), responsible for acute hepatitis in humans, is an RNA virus belonging to the family *Hepeviridae* and the subfamily *Orthohepevirinae*, and can be classified into four genera. The *Paslahepevirus* genus comprises the *Paslahepevirus balayani* species, including the zoonotic HEV-3 and HEV-4 genotypes, causing sporadic cases in high-income countries, with pigs and wild boars as the main reservoirs. The *Rocahepevirus* genus includes two species: *Rocahepevirus eothenomi* reported in Chevrier’s Field Mouse (*Apodemus chevrieri*) and Pere David’s Vole (*Eothenomys melanogaster*), and the *Rocahepevirus ratti* (rat-HEV) species, which is divided in two genotypes (HEV-C1 and -C2). HEV-C1 has been detected in rats (*Rattus* sp.; *Bandicota indica*) and eulipotyphlans (musk shrew, *Suncus murinus*), while HEV-C2 has been detected in mustelids (ferret and mink) [1]. Initially identified in Germany [2], HEV-C1 has been detected in Europe, Asia and North America in several species of rats [3]. There is existing evidence of HEV-specific antibodies in multiple rat species, including *Rattus rattus* (black rat) and *Rattus norvegicus* (brown rat) [2,4,5,6,7,8,9].

The HEV-3 strains have been sporadically reported in brown and black rats, exclusively in the intestines and feces, and never in the liver, suggesting ingestion and passive transport of feces containing HEV-3 rather than an actual infection [10].

In addition to a passive transit of HEV-3 in the intestine, infection and a localized digestive replication in rats could also occur, as suggested by a few studies. The isolation of infectious HEV-3 in the spleen and intestines in rats without any sign of replication in the liver, suggests that HEV could infect these organs and replicate in rats [11]. The hypothesis that HEV-3 can replicate in the mucosal of intestine has also been confirmed in vitro on cell cultures of HEV-1 and HEV-3 [12].

In recent years, HEV-C1 strains belonging to the *Rocahepevirus ratti* species (HEV-C1), previously described as being exclusively hosted by rats, have been reported in 21 human patients with acute or chronic hepatitis. The 21 HEV-C1 infections have been confirmed through RNA detection in human specimens in Hong Kong [13,14,15], Canada [16], France [17] and Spain [18], while in Germany no rat- HEV infections were reported in a retrospective study [19]. The presence of rat-HEV-RNA and IgG and IgM antibodies against HEV-C1 in patients confirmed the infection [3]. Antibodies against HEV-C were also detected in healthy forestry workers, suggesting a risk linked to their exposure to rat excrements [20]. The reported studies evidence that HEV-C1 can infect humans, with rats serving as source of infection, and suggest that an underestimation of the number of cases could be possible [20,21]. However, the zoonotic transmission of the HEV-C1 genotype is still controversial. The specific pathway of human infection transmission remains unclear; however, various potential risk factors have been suggested, including exposure to polluted environments, interaction with infected animals or the consumption of food or water that may be contaminated [3].

In Italy, HEV-C1 and HEV-3 were detected in black and brown rats [10,22] and in wastewater samples collected in Central Italy [23]. In one of the studies, rats were sampled from the area surrounding a farm housing HEV-3-infected pigs, suggesting the possible ingestion of feces from HEV-infected pigs [10]. Interestingly, a recent study in Spain reported the detection of rat-HEV strains in the rectal fecal samples of 44 pigs out of 387 investigated. This suggests that pigs could also have a role in the epidemiology of rat HEV-C1 and vice versa [24].

In this study, our aim was to identify and characterize HEV strains circulating in rats in Northeast Italy in urban and peri-urban areas not investigated before to assess the role of this species in the epidemiology of HEV.

## 2. Materials and Methods

### 2.1. Samples Collection and RNA Extractions

During 2020–2023, 70 brown rats (*Rattus norvegicus*) and 45 black rats (*Rattus rattus*) were collected from urban and peri-urban areas of Northeast Italy (area 4156 km^2^), as part of pest control programs. Necropsy was performed with sterile instruments, and depending on the condition of the carcasses, 79 livers, 92 lungs and 103 intestinal contents were sampled. Fecal samples were stored at −20 °C and organs at −80 °C.

Total RNA was extracted from 200 µL of 10% fecal suspension (g/vol) obtained from intestinal contents using the QIAamp Viral RNA Kit (Qiagen, Hilden, Germany) and from livers and lungs (25 mg) using the RNeasy Mini Kit (Qiagen, Hilden, Germany) following the manufacturer’s instructions. Positive control HEV-3-positive feces and a negative control (water) were also included. The 25 mg were previously homogenized in lysis buffer available in the kit, using the Tissue lyser (Qiagen, Hilden, Germany). The RNA extracted was stored at −80 °C until use.

### 2.2. HEV RNA Detection by Real-Time and Conventional Reverse Transcription PCR

All RNAs were tested for the detection of the genotypes of *Paslahepevirus balayani* species (including HEV-1 to HEV-4 genotypes) and HEV-C, as described below. The RNA samples were tested for the detection of the former genotypes with broad-range real-time reverse transcription PCR (real-time RT-PCR) using the QuantiFast Pathogen RT-PCR +IC Kit (Qiagen, Hilden, Germany) [25], the kit included the internal control (IC); a second test was a conventional reverse transcription PCR (RT-PCR, QIAGEN OneStep RT-PCR Kit; Qiagen, Hilden, Germany) followed by nested PCR (Go Taq, Promega, Madison, WI, USA) using the broad range HEV-1-4 test amplifying a 400 bp region within the ORF2 [10]. A second pan-*Hepeviridae* test, able to amplify *Paslahepevirus balayani* and the HEV-C, was performed to amplify a 300 bp within the RNA-dependent RNA polymerase region (RdRp) of the ORF1 [26]. Those samples positive for the latter RT-PCR were also subjected to additional RT-PCRs using primer pairs specifically aligned to the HEV-C1 [27] to obtain amplicons of nearly complete genomes (Supplementary Table S1). The internal control, IC, included in the kit for the real-time RT-PCR was used to discard the presence of PCR inhibitors in the RNA. A positive control in the form of HEV-3 genotype and negative controls were included in all steps, from RNA extractions to PCRs [10]. PCRs were stored at −20 °C until use or were immediately run using agarose gel electrophoresis (1.5%).

### 2.3. Sequencing and Phylogenetic Analyses

The positive PCR products were cleaned up with ExoSAP PCR Enzymatic Clean-Up Kit (Euroclone, Pero, Italy). Amplicons of expected size were sequenced by Sanger sequencing through a custom sequencing service (Eurofins Genomics, Ebersberg, Germany). Sequences were uploaded to the GenBank NCBI database under the following accession numbers: PP067025-PP067033 (RdRp), PP827493-PP827494 (methyltransferase), PP827495-PP827496 (3′-ORF1 to 3′-ORF2). The nucleotide sequences were edited and aligned using the Aliview free software ver. 1.28 [28]. The related sequences were searched using the BLASTn server on the NCBI GenBank database (http://www.ncbi.nlm.nih.gov/genbank/index.html, accessed on 2 May 2024) and the maximum likelihood (ML) trees were drawn with IQTREE2 software ver. 2.0 [29] using the model suggested by the model test and 1000 bootstrap replicates.

### 2.4. Capsid Proteins Alignment

Analysis of the capsid protein amino acidic sequences and the prediction of its secondary structure was performed using SWISS-MODEL (https://swissmodel.expasy.org, accessed on 2 May 2024) based on default parameters. Secondary structure elements were highlighted using the ESPript (http://espript.ibcp.fr, accessed on 2 May 2024) algorithm [30] and compared to capsid protein from representative strains of HEV-3 and HEV-C1 (subtype G1–G3).

## 3. Results

None of the samples tested positive for any of the *Paslahepevirus balayani* genotypes, including the HEV-1 to HEV-4, in either the broad range real-time RT-PCR or the conventional RT-PCR. However, the RNAs from three animals tested positive (3/115, 2.6%) by the pan *Hepeviridae* nested RT-PCR, amplifying the RdRp fragment, in paired intestinal contents (3/103, 2.9%), livers (3/79, 3.8%) and lungs (3/92, 3.3%). The positive rats, two brown rats and one black rat, were adult females that were sampled in 2021 from two municipalities. The sequences obtained from the feces, lungs and liver of each animal were identical (100% nucleotide identity; nt.id.).

Sequence analyses of the RdRp fragments, which were identical across each rat, showed that the RatHEV115IT21 sequences exhibited 98.1% nt.id. with RatHEV118IT21, while RatHEV119IT21 showed an 80.0% nt.id. with RatHEV115IT21 and 82.0% with RatHEV118IT21.

**Figure 1:**
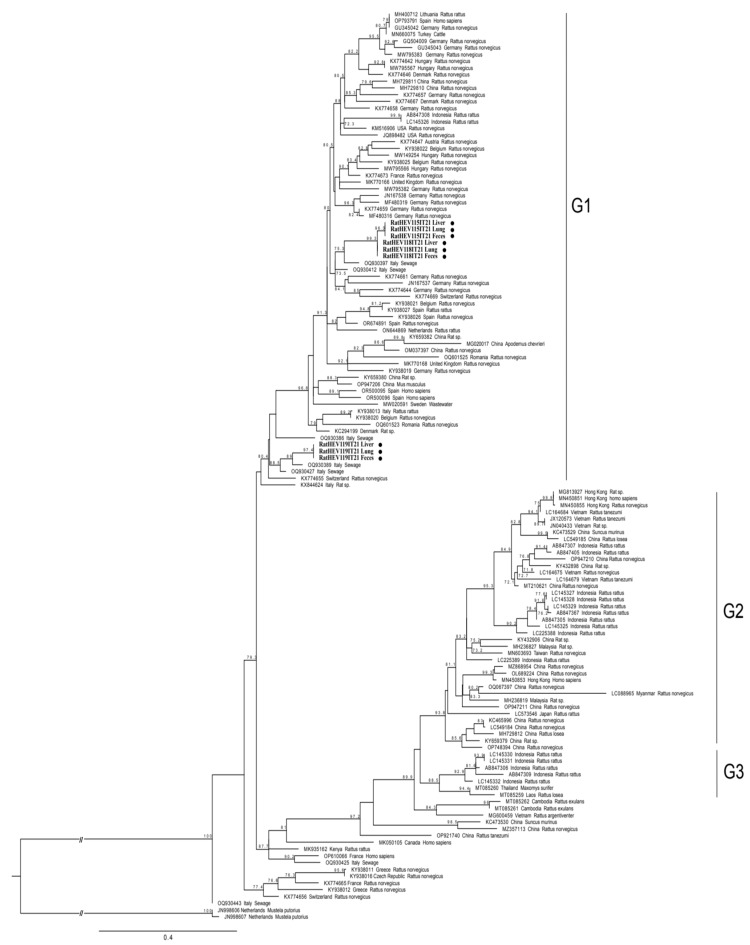
Phylogenetic analysis based on the 270 nt fragment of the partial RdRp region within ORF1 of the 9 sequences obtained in this study (entries highlighted in bold and indicated by black dots), 124 HEV-C1 sequences obtained from NBCI database by BLASTn searches, and two HEV-C2 sequences used as an outgroup. The maximum likelihood tree was produced using the TIM2 model (Transition model 2) with invariant sites and gamma distribution based on 1000 bootstrap replications and bootstraps values >70 indicated at their respective nodes. Sequence entries are reported as GenBank Accession Number, Country and Host species. On the right side, sequences belonging to G1–G3 group of HEV-C1 are indicated.

Since the majority of rat HEV sequences submitted to the NCBI database were obtained by amplifying the RdRp region within ORF1, a phylogenetic tree was first built based on this region (Figure 1). The nine sequences obtained in this study from animal RatHEV115IT21, RatHEV118IT21 and RatHEV119IT21 were classified as an HEV-C1 genotype within group GI (Figure 1), which is one of the three (GI-GIII) groups of putative subtypes proposed within the HEV-C1 strains based on phylogenetic analysis [31].

Compared to other HEV-C1 strains detected worldwide, the three sequences in this study formed distinct clusters (Figure 1), and in the sequence alignment, showed <89.0% nt.id. with the other sequences. The nine sequences of RdRp, three of which were identical in each animal, from this study clustered together (Figure 1) with sequences detected in sewage in Central Italy [23] in 2020 (Figure 1, entries named in the tree as “Italy Sewage”), displaying an nt.id, in the RdRp sequences that ranged between 90.4% and 92.6%. The two strains RatHEV115IT21 and RatHEV118IT21 were closely related (98.1% nt.id.) to each other, clustered together and were both identified in black rats captured in two provinces 60 km apart, while the RatHEV119IT21 sequence which was detected in a brown rat in another area, clustered separately, as shown by the phylogenetic analyses (Figure 1).

In detail, the RatHEV115IT21 and RatHEV118IT21 strains showed 90.4–90.7% nt.id., respectively, with the 3785-20/CH/IT sequence detected in sewage (GenBank Accession Number: OQ930397.1), while RatHEV119IT21 showed a 92.6% nt.id. with both 4361-21/CH/IT (OQ930427.1) and 3766-20/CH/IT (OQ930389.1) which were also detected in sewage in Italy.

The HEV-C1 strain identified in 2015 in a *Rattus rattus* in Italy (Rat/ITA/4/ITA/2015, KY938013.1) shared 86.2%, 86.1% and 84.4% nt.id. with RatHEV115IT21, RatHEV118IT21 and RatHEV119IT21, respectively. Additionally, sequences from this study showed 82.5%, 82.8% and 85.8% nt.id., respectively, with another Italian rat strain, RatHEV18IT16 (KX844624.1). The rat-HEV, Rat/ITA/4/ITA/2015, was detected in a black rat (*Rattus rattus*) on a small island [22] and RatHEV18IT16 was retrieved from a black rat captured in an area surrounding pig farms [10]. Nevertheless, a higher nucleotide identity of the three rat strains from this study was observed with sequences retrieved by analyzing wastewater samples from Central Italy (Abruzzo region) (OQ930397, OQ930412, OQ930389, OQ930427) [23] than with the RatHEV18IT16 (KX844624.1) strain previously reported in a rat captured in the same Italian region [10]. The lower correlation was confirmed in the phylogenetic tree based on the RdRp fragment (Figure 1); the two strains previously identified in rats in Italy (KY938013.1 and KX844624.1), clustered separately from the three strain sequences in this study.

To deeply analyze the genome sequences of the Italian rat-HEV-C1 strains, additional genome regions of interests were sequenced. Specifically, the methyltransferase within ORF1, position 50–942 nt with respect to the reference with the RefSeq Accession number NC_038504.1 (rat/R63/DEU/2009, GenBank Accession Number GU345042), and 3934 nt, encompassing the 3′ terminal of the ORF1 to the 3′ terminal of the ORF2, (position 2890–6824 nt reference rat/R63/DEU/2009) were obtained from the feces of RatHEV115IT21 and RatHEV119IT21.

Despite several attempts, we did not succeed in sequencing RatHEV118IT21, probably due to the low amount of RNA, and the full genome was not obtained for any of the samples. The phylogenetic analysis based on longer sequences (3934 nt encompassing the 3′ terminal of ORF1 to 3′ terminal of the ORF2) confirmed the classification of both strains as HEV-C1 genotype within group GI, as previously observed by the analysis of short genome fragment of the RdRp (position 4124–4352 nt reference rat/R63/DEU/2009) (Figure 1).

The group in which the rat strains identified in the present study cluster does not include any strains that have been detected in humans.

The rat HEV-C1 strains identified have shown an approximately 84–85% shared nucleotide identity within the short RdRp genomic region with two human strains reported in Spain (OK082152, OK082153). When comparing longer sequences, the closest match among the RatHEV115IT21 and RatHEV119IT21 strains was a French strain (22072190255_HEV-C, OP610066) with approximately the same identity as that of the shorter fragment with 84.7% and 84.6% shared nucleotide identities, respectively.

Based on SWISS protein research, the protein displaying the more similar amino acid (aa) identity to the HEV rat capsid proteins from this study was the partial capsid protein from a HEV-3 strain (amino acids 112–608; PDB Accession Number: 2ZTN) since no crystals of complete capsid protein from HEV-C1 strains are available online.

**Figure 2:**
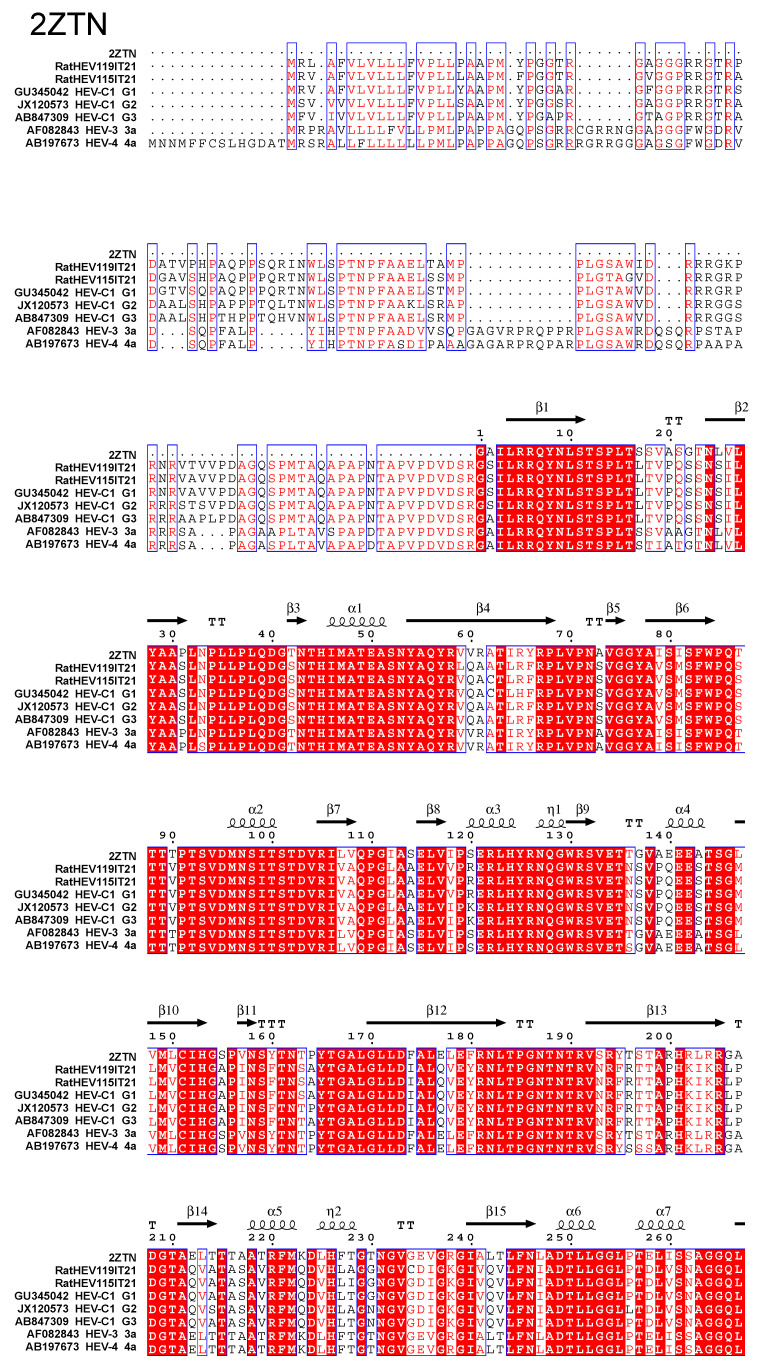

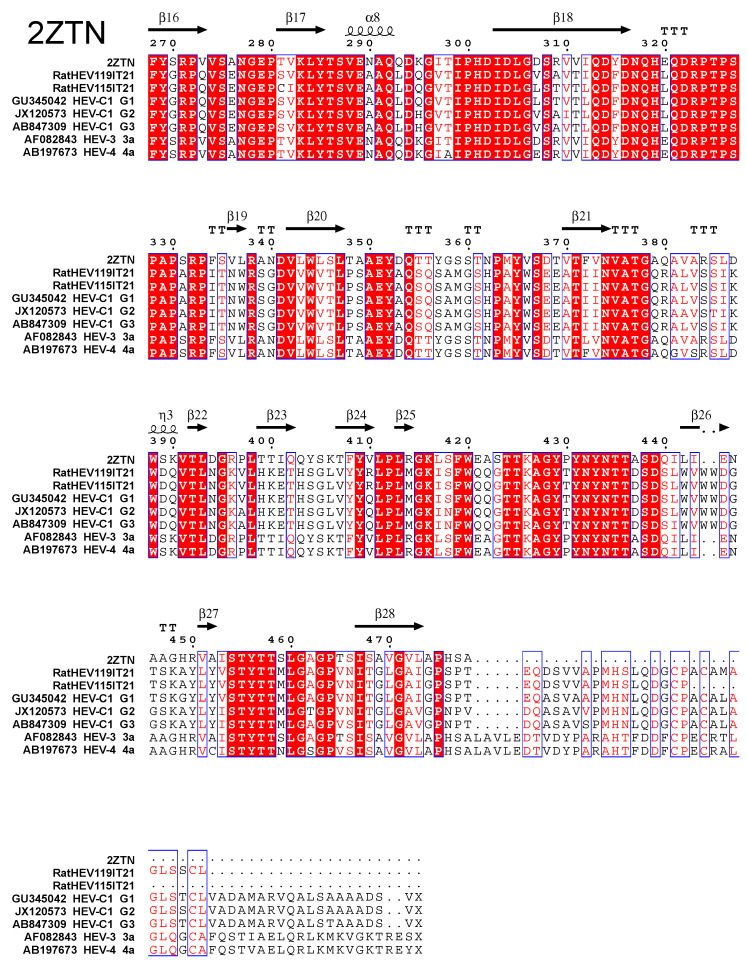
Alignment of amino acid sequences and secondary structure elements of HEV capsid proteins. The first line indicates the PDB number (2ZTN) of the capsid protein from the HEV-3 strain along with its secondary structure elements. On the left, the names of strains analyzed in the capsid proteins are indicated with accession numbers, genotypes, and subtypes. The capsid protein of the strains sequenced in this study are indicated by the names RatHEV119IT21 and RatHEV115IT21. Spiral lines indicate helices, while arrows represent β strands. White characters in red boxes represent strictly conserved residues and red characters represent stereochemically identical residues.

To predict the possible secondary elements, we analyzed the alignment of the capsid protein amino acid sequences between the rat HEV-C1 strains and other HEV strains (Figure 2). The RatHEV119IT21 and RatHEV115IT21 strains shared 95.5% aa.id. with each other. The RatHEV119IT21 capsid protein had a 95.5% aa.id. with reference strains belonging to the rat HEV-C1 group G1 (GU345042; from *Rattus norvegicus*), 93.1% with G2 (AB847309; from *Rattus rattus*) and 92.8% with G3 (JX120573, from *Rattus tanezumi*).

The capsid of RatHEV115IT21 had a 97.3% aa.id. with HEV-C1 G1 (GU345042), qhich was higher than with the RatHEV119IT21 (95.5%), and approximately 96% with HEV-C1 G2 and G3 (AB847309, JX120573). Both Italian rat-HEV strains showed a significantly lower aa.id. of 62–63.2% with the HEV-3 and HEV-4 strains (AF082843, AB197673).

Comparing the aa within the three domains of the capsid protein, the shell (S) (aa 129–319), the middle (M) (aa 320–455) and the protruding (P) domains (aa 456–606), the rat HEV-C1 strains displayed more similar amino acid identities in the S and M domains with the HEV-3 and HEV-4 strains, while the shared identity significantly decreased to 46% aa.id. in the P domain.

The alignment of the RatHEV119IT21 and RatHEV115IT21 capsid proteins with the HEV-3, HEV-4 and HEV-C1 strains (Figure 2) revealed that nine out of the thirty-nine secondary elements had identical amino acids (comprising five beta sheets and four alpha helices). Additionally, seven elements (four beta sheets and three alpha helices) showed only one amino acid difference between the HEV-C1 strains and HEV-3/4 strains. In one element (Beta3), the substituted amino acid had similar chemical properties (e.g., serine in HEV-3/4 strains and threonine in HEV-C1 strains).

At the 5′ of the ORF1, which partially overlapped with the region coding for methyltransferases both strains showed the presence of the hypothetical ORF coding for the protein ORF4 (position 50–578 with respect to reference rat/R63/DEU/2009, NC_038504.1). The ORF4 of both HEV-C strains of this study shared an 83.9% aa.id. Both Italian strains had the highest aa.id. with two HEV-C1 strains reported in Korea from *Rattus norvegicus*: RatHEV119IT21 had an 85.1% aa.id. with the Rn19-14 strain (OR500096) and RatHEV115IT21 had 83.9% aa.id. with Rn16-10 (OR500095).

## 4. Discussion

Overall, our data showed a 2.6% positivity for HEV-C1 RNA in the rat population analyzed, confirming its presence in various rat species. The circulation of HEV-C1 in rats has previously been reported in Germany (2.9%) [5], Romania (17.4%) [9], China (1.7%) [32], Japan (1.2%) [8] and Korea (4.4%) [33]. In a survey conducted in 11 countries in Europe, rat-HEV was detected with a prevalence ranging from 4.0 to 41.3% in both rural and urban rats [22]. The results are difficult to compare, but the prevalence revealed in this study may be considered low when compared to Romania (17.4%) [9] but comparable to Germany (2.9%) [5] and to previous findings in black rats from Italy (2.1%) [10]. In this study, compared to the previous studies on rats in Italy [10], the animals investigated were from urban and peri-urban areas, in locations closer to human environments, including its wastewater and garbage, which highlights risks for humans associated with the HEV-C1.

The results of a comparison with other Italian HEV-C1 sequences available online at the NCBI showed a high diversity of rat-HEV strains circulating in Italy, consistent with findings from sequences obtained in 14 wastewater plants in one region that shared 82.5–95.8% nt.id. with each other [23]. The sequence comparison was limited to the RdRp fragment, since other rat HEV-C1 strains detected in Italy were only sequenced in this short region. By comparing the longer sequence stretch (3934 nt, 3′-ORF1 and the whole ORF2)) with the NCBI database and constructing a phylogenetic tree, we confirmed the classification within the HEV-C1 group GI. The results showed that the RdRp sequence analysis closely resembles that obtained with the longer stretch. Only a limited number of HEV-C1 sequences are available at the NCBI, aside from the RdRp fragment, which limits further analysis. However, the phylogenetic analysis confirmed that HEV-C1 in this study clustered with other Italian sequences retrieved from the wastewater samples, even displaying a limited shared nucleotide identity with them [23].

The detection of the same rat-HEV strain in paired intestinal contents, livers and lungs suggests active HEV replication and excretion. The detection of HEV-RNA in the lungs of animals with positive findings in the liver and feces was reported in two studies [34,35]. The authors retrieved rat-HEV RNA from the lungs, kidneys and hearts of rats that tested positive for rat-HEV RNA in the liver and blood. Other studies also reported rat-HEV in feces, liver and sera [36,37]. The lung is one of the most important vascularized organs in rats [38], suggesting that the presence of HEV-RNA could be associated with viremia, but replication in the lungs cannot be excluded and deserves further investigation. Extrahepatic manifestations of the disease associated with the lungs in humans have not been described and further studies could be performed to investigate other organs involved in the replication of the HEV [39] in rats as well as other animal hosts. In this study, HEV-3 was not identified. Conversely, in our previous study, the intestine contents of a rat captured in an area surrounding pig farm were HEV-3 positive [10]. The presence of this genome in rats could be considered either accidental and probably linked to ingestion of pig feces contaminated with HEV-3, or due to replication in the intestine which could also occur [11]. This hypothesis needs to be investigated if the replication of HEV-3 or HEV-C strains in the rat intestine is confirmed as it could serve as a major route for virus dissemination in the environment.

The analysis of the amino acid alignments of the capsid proteins showed the Italian rat strains had highly similar amino acids identities with the HEV-C1 strain subtype G1, confirming the results obtained from the nucleotide identity tests. Furthermore, the results revealed higher conservation in the S and M domains between HEV-C and HEV-3, suggesting that they are key structural elements in viral particles, potentially indicating a conserved assembly process for HEV.

The low similarity in the amino acid identity between rat HEV-C strains and *Paslahepevirus balayani* species strains in the P domain confirms previous findings [2] suggesting that divergence in the protruding parts of the outer capsid surface, containing the neutralization epitopes, may reflect differences in HEV recognition and host–cell entry among species [40,41].

The role of epitopes mapped to the P-domain is confirmed by several studies, monoclonal antibodies (mAbs) directed against rat-HEV mapping the P-domain did not show cross-reactivity with human HEV ORF2 (genotypes 1, 3 and 4) and mAbs, which is able to neutralize human HEV (genotypes HEV-1, HEV-3, and HEV-4) [42], did not exhibit cross-reactivity with rat HEV.

Nevertheless, the stretch of aa “ADTLLGGLPTELISSA” within the P-domain, identified as a potential polysaccharide-binding site that may function in cell-receptor binding, is strictly conserved among all HEV1-4 genotypes [40], was previously identified in rat HEV-C1 strains [36] and in the strains investigated in this study.

In this study, the presence of ORF4 within the ORF1 in HEV-C1 strains has been confirmed, as previously reported in HEV-C strains from rats [2,27,43,44]. The function of ORF4 is unknown [45]. The ORF4 protein was not detected in the liver tissue of HEV-infected rats and the protein is not necessary for viral replication [46].

The number of studies reporting rat HEV-C1 in humans has been increasing but the role of rats as possible reservoirs of zoonotic strains remains unknown. Rats pose a notable potential reservoir for HEV strains with the potential to infect humans, as confirmed by the detection of human cases linked to rat HEV-C1 and by the presence of similar capsid structures between rat-HEV and host-specific human HEV. Although rats are not a regular part of the human diet, and under appropriate hygiene conditions they should ideally be kept separate from humans, their population is increasing significantly in cities and they show a high anti-HEV seroprevalence [47]. Their potentially contaminated feces can accumulate in wastewater and be released into the environment. Furthermore, despite pest control efforts, rats may come into contact with naturally virus-hosting animals such as pigs or wild boar. We are not fully aware of the consequences of HEV-C1 being released in the environment and contaminating food products like vegetables if they are consumed without proper washing. All of these points together with detection of HEV-C1 human infections underscore the importance of understanding potential health risks and suggest that further investigations are crucial to evaluate the role of rats as zoonotic hosts of HEV and clarify the epidemiology of circulating strains.

## Data Availability

Sequences obtained from this study have been deposited at GenBank under the accession PP067025–PP067033; PP827493–PP827496.

## Supplementary Materials

The following supporting information can be downloaded at: https://www.mdpi.com/article/10.3390/pathogens13080633/s1, Table S1: List of primers used to amplify the HEV-C near-full-length genome [27,48].
