# Supplementary material for: Investigating the Hepatitis E Virus (HEV) Diversity in Rat Reservoirs from Northern Italy
Source: Pathogens. 2024 Jul 29;13(8):633. doi: 10.3390/pathogens13080633 (PMC11357196; doi:10.3390/pathogens13080633)
Supplement: Supplementary file 1 [file pathogens-13-00633-s001.zip › pathogens-3128562-supplementary.pdf]

Table S1. List of primers used to amplify the HEV-C near-full-length genome

Primer	Orientation	Sequence (5'-3')	Genomic Position *	Length of Target Fragment, bp
**HEV-F15**	Sense (first/second round)	AGACCCATCARTATGTCG	15	921 [27]
**HEV-R935**	Antisense (second round)	GTDGAYCKRGMCTTCTCACA	935	
**HEV-R977**	Antisense (first round)	CATRAGYCKRTCCCADAT	977	
**HEV-F2868**	Sense (first/second round)	TKAARGCNCARTGGMGDG	2868	841 [27]
**HEV-R3708**	Antisense (second round)	ARBAYDGTCACCTGVTCHC	3708	
**HEV-R3740**	Antisense (first round)	ATRCGRCARTGCACDGT	3740	
**HEV-F3539**	Sense (first/second round)	ACCAACTTGCAGGATATAG	3539	699 [27]
**HEV-R4237**	Antisense (second round)	AAACTCGCTAAAATCATTCTCAA	4237	
**HEV-R4253**	Antisense (first round)	ATTCTGGGTGCTGTCAAACTCG	4253	
**HEV-F4980**	Sense (first/second round)	TCGTGCTCGTGYTTTTGCT	4980	1029 [27]
**HEV-R6008**	Antisense (second round)	CCTATRTCRCCYACMCCRTT	6008	
**HEV-R6014**	Antisense (first round)	CCCTTRCCTATRTCRCCYAC	6014	
**HEV-F5552**	Sense (first/second round)	GTRTCAATGTCRTTYTGG	5552	1153 [27]
**HEV-R6704**	Antisense (second round)	RTTAACAGGYCCAGYACC	6704	
**HEV-R6836**	Antisense (first round)	ATWGCATCAGCMACGAGGCA	6836	
**HEV-F6300**	Sense (first/second round)	CAACTGGCGGTCTGGTGATGTC	6300	535 [27]
**HEV-R6834**	Antisense (second round)	AGACACTGTCGGCTGCTGC	6834	
**HEV-R6881**	Antisense (first round)	GCATCAGCCACGAGGCAGG	6881	
**HE607**	Sense (first round)	CTTGGTTYAGGGCCATAGAG	4098	880 [48]
**HE604**	Antisense (first round)	CAGCAGCGGCACGAACAGCA	4977	
**HE608**	Sense (second round)	TTYAGGGCCATAGAGAAGGC	4103	
**HE606**	Antisense (second round)	ACAGCAAAAGCACGAGCACG	4963	
**RatFw3570**	Sense (first/second round)	CCATAGACCGCTTGAGGTG	3601	550<br>Designed in this study
**RatRw4120**	Antisense (second round)	AGCAATCGCCGTAGAAACAC	4166	
**RatRwN4160**	Antisense (first round)	CGTCCACGATGGCTTTCT	4133	

* respect to the reference NC_038504.1 (RefSeq Accession Number)
